# A new *Stenoloba* Staudinger species from China (Lepidoptera, Noctuidae, Bryophilinae)

**DOI:** 10.3897/zookeys.108.1208

**Published:** 2011-06-17

**Authors:** Oleg Pekarsky

**Affiliations:** 1 H-1068 Budapest, Felsőerdősor u. 16-18, Hungary

**Keywords:** Lepidoptera, Noctuidae, *Stenoloba*, new species, China

## Abstract

A new species of *Stenoloba*, *Stenoloba viridicollar* **sp. n.** (Lepidoptera, Noctuidae) is described from Sichuan, China. Illustrations of adults and the genitalia of both sexes are provided. A diagnostic comparison is made with *Stenoloba rufosagitta* Kononenko & Ronkay, 2001 *and S. rufosagittoides* Han & Kononenko, 2009.

## Introduction

*Stenoloba* Staudinger, 1892 is an East Asian genus of the subfamily Bryophilinae. Itwas included in the subfamily Acontiinae (sensu auctorum) by early authors until Sugi (1970) revised the Japanese species of the genus and established its position in the Bryophilinae. The East Asian fauna of the genus was revised by (Kononenko and Ronkay (2000, 2001). Chen (1999) only listed seven species of *Stenoloba* from China. A complete review of Chinese *Stenoloba* was published by Han and Kononenko (2009), listing 37 species known to occur in China. The genus presently includes, according to the last remarkable contribution on the taxonomy of the genus (Behounek and Kononenko 2010), 75 species which are arranged in 14 species groups by Behounek and Kononenko.

During a study of the Chinese *Stenoloba* material collected by Viktor Sinyaev in Sichuan province in 2008, it was surprising to find that the *Stenoloba glaucescens* species-group contains another undescribed, species. The new species, described below, externally resembles *Stenoloba rufosagitta* and *Stenoloba rufosagittoides*, but has clearly recognisable differences in its external and genital features.

## Systematic part

### Stenoloba viridicollar

Pekarsky sp. n.

urn:lsid:zoobank.org:act:DBF5569D-E216-4C8B-8A41-7DE6CB32DA69

http://species-id.net/wiki/Stenoloba_viridicollar

Figs 1– 4 

#### Holotype.

Male, China, Sichuan, Lao Lin Kou, 1900m, 28°21’N, 103°26’E, 26.vi.–12.vii.2008, leg. Viktor Sinyaev; slide No.: OP1034m (coll. O. Pekarsky, deposited in the HNHM Budapest).

#### Paratype.

China, Sichuan: 2 males, 2 females, with same data as for holotype (coll. O. Pekarsky).

#### Diagnosis.

The new species is externally similar to *Stenoloba rufosagitta* and *Stenoloba rufosagittoides*, combining characteristics of both relatives. The most prominent distinguishing feature is the greenish-grey colouration of the head and collar, which are grey brown to dark brown in the two related species. In addition, *Stenoloba viridicollar* differs from *Stenoloba rufosagitta* by the more oblique and less arched outer margin and more unicolorous pattern of the forewing. It differs from *Stenoloba rufosagittoides* by its broader forewing with a wider marginal area. The basal area of the new species is less marked then in the other two species. The specific features of the male genitalia are the shape of the valva, the shape and size of the the ampulla, and the structure of the vesica. The most conspicuous autapomorphy of *Stenoloba viridicollar* is the rather short triangular valva with more or less straight costal and ventral margins, and the acutely triangular cucullus without a hooked tip or subapical process but with a long, narrow ampulla. The new species is easily distinguishable from the related two species because *Stenoloba rufosagitta* has a long, rod-like valva with more or less parallel costal and ventral margins, a somewhat rounded cucullus with fine, short, hooked tip, and the ampulla is missing; *Stenoloba rufosagittoides* is characterized by the long, hooked, rather claw-like apical saccular extension and the asymmetrical subapical costal processes.

#### Description.

**Figures 1–2.**
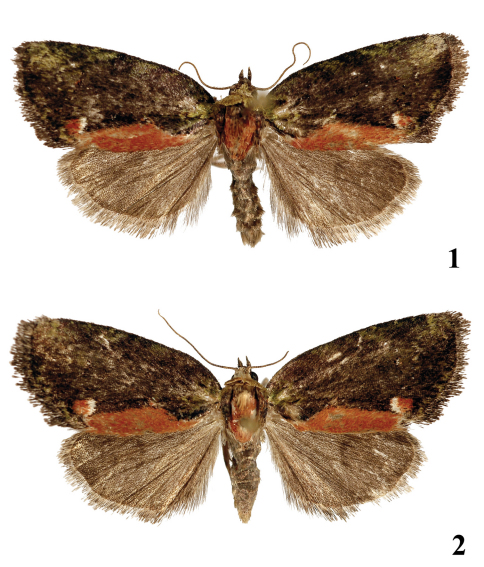
Adults. **1** *Stenoloba viridicollar* sp. n., holotype male, China **2** *Stenoloba viridicollar* sp. n., paratype female, China.

Male. (Fig. 1). Wingspan 22 mm. Head and collar greenish grey, thorax blackish grey, with rufous mesothorax and blackish-grey tegulae mixed sparsely with rufous scales; abdomen blackish grey. Forewing relatively short, slightly dilated towards outer edge; costa arched basally; apex finely pointed; outer margin more oblique and straighter than in *Stenoloba rufosagittoides* and even more so than in *Stenoloba rufosagitta*.Ground colour of forewing blackish brown, wing pattern diffuse, less traceable than in *Stenoloba rufosagittoides*; basal field and costal area with small greenish patches between crosslines; crosslines dark blackish grey, basal line relatively strongly marked; subbasal line defined indistinctly by green scales; antemedial line S-shaped, obsolescent; medial area somewhat darker than ground colour; postmedial line undulate; subterminal line present but indistinct; typical noctuid maculation hardly recognisable, reniform stigma somewhat more sharply defined with some black and rufous spots; inner margin with conspicuous rufous “pirate sword”-shaped stripe extending from base of wing to postmedial line; tornal patch rufous with white scales inside and with black scales outside; termen suffused with green; cilia as for ground colour. Hindwing uniformly dark brown, discal spot hardly traceable. Female. (Fig. 2) as for male but somewhat larger in size (wingspan 24 mm), with less expressed forewing pattern.

#### Male genitalia.

**Figures 3–5.**
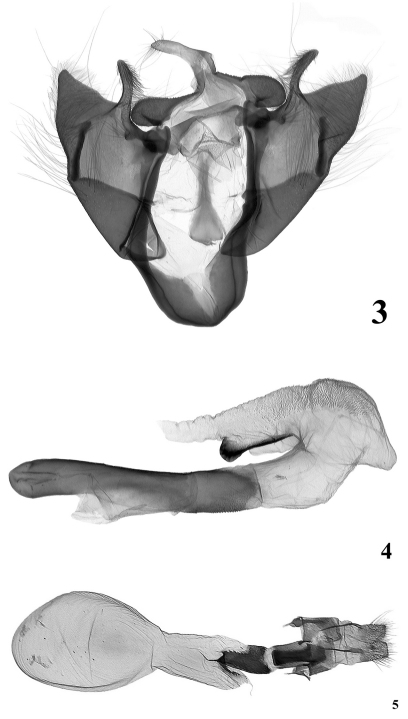
Genitalia **3, 4** *Stenoloba viridicollar* sp. n., male genitalia, holotype, China, slide No. OP1034m **5** *Stenoloba viridicollar* sp. n., female genitalia, paratype, China, Slide No. OP0369f.

(Figs 3, 4). Genital armature with opened valvae looks like an equilateral triangle; all structures heavily sclerotized. Uncus short and strong, triangular and flattened; tegumen two times shorter than vinculum; penicular lobes large, verrucose; juxta long, deltoidal, with rounded basal (ventral) side and long, thin apical (dorsal) extension; vinculum with saccus wide and massive; valvae symmetrical, massive, shortly triangular with very wide base and short apical section with straight sides; sacculus rather narrow, short, covered by small pimples from middle; costa very short; ampulla long, slender and arcuate, bearing relatively short bristles; editum with 12 long bristles. Aedeagus relatively long and thin, bulbus ejaculatorius wide, carina covered by fine denticles. Vesica tubular, everted posteriorly, then bent ventrad and recurved along ventral side of aedeagus. Basal tube membranous, medial section dilated and inflated, rather globular, with short subconical frontal diverticulum and somewhat longer but thinner ventro-lateral diverticulum. Ventral surface of medial section and distal tube densely scobinate and finely spinose; dorsal side of distal tube with large, sclerotised crest-like cornutus.

#### Female genitalia.

(Fig. 5). Ovipositor short, conical; apophyses anteriores short and thin, wide based; apophyses posteriores relatively long and stout, four times longer then apophyses anteriores. Antrum long, wide, heavily sclerotized; ductus bursae similarly sclerotised, with narrow membranous ring (“neck”) between antrum and ductus bursae; appendix bursae semiglobular-subconical, membranous; corpus bursae with narrower posterior part and elliptical-ovoid proximal section.

#### Note.

It is worth mentioning that the two other species in the *Stenoloba rufosagitta* group were described only from males, so the identification of the female of the new species is based on external features. The characteristic greenish colouration of the head and collar corresponds well with those of the male and differs prominently from that of the two related taxa. Moreover, all moths of the type series were collected in the same site and date and no other *Stenoloba* species were found together with them.

#### Etymology.

The name “*viridicollar*” refers to the greyish-green coloration of head and collar, which is the main external distinguishing character of the species.

#### Distribution.

The species is known only from the type-locality, South-West China, Prov. Sichuan, Lao Lin Kou.

## Supplementary Material

XML Treatment for Stenoloba viridicollar
